# Development of a nomogram to predict the risk of hepatocellular carcinoma in patients with hepatitis B-related cirrhosis on antivirals

**DOI:** 10.3389/fonc.2023.1128062

**Published:** 2023-02-16

**Authors:** Ke Shi, Ping Li, Qun Zhang, Yi Zhang, Yufei Bi, Xuanwei Zeng, Xianbo Wang

**Affiliations:** ^1^ Center of Integrative Medicine, Beijing Ditan Hospital, Capital Medical University, Beijing, China; ^2^ Department of Hepatology, Tianjin Second People’s Hospital, Tianjin, China

**Keywords:** hepatitis B virus, hepatocellular carcinoma, cirrhosis, prediction model, risk stratification

## Abstract

**Objective:**

Patients with compensated hepatitis B-related cirrhosis receiving antivirals are at the risk of hepatocellular carcinoma (HCC). This study aimed to develop and validate a nomogram for predicting the incidence of HCC in patients with hepatitis-B related cirrhosis.

**Design:**

A total of 632 patients with compensated hepatitis-B related cirrhosis treated with entecavir or tenofovir between August 2010 and July 2018 were enrolled. Cox regression analysis was used to identify independent risk factors for HCC and a nomogram was developed using these factors. The area under the receiver operating characteristic curve (AUC), calibration curve, and decision curve analyses were used to evaluate the nomogram performance. The results were validated in an external cohort (n = 324).

**Results:**

In the multivariate analysis, age per 10 years, neutrophil–lymphocyte ratio > 1.6, and platelet count < 86×10^9^/L were independent predictors of HCC occurrence. A nomogram was developed to predict HCC risk using these three factors (ranging from 0 to 20). The nomogram showed better performance (AUC: 0.83) than that of the established models (all *P* < 0.05). The 3-year cumulative HCC incidences in the low- (scores < 4), medium- (4–10), and high-risk (> 10) subgroups were 0.7%, 4.3%, and 17.7%, respectively, in the derivation cohort, and 1.2%, 3.9%, and 17.8%, respectively, in the validation cohort.

**Conclusion:**

The nomogram showed good discrimination and calibration in estimating HCC risk in patients with hepatitis-B related cirrhosis on antivirals. High-risk patients with a score > 10 points require close surveillance.

## Introduction

Hepatitis B virus (HBV) is the leading cause of cirrhosis and hepatocellular carcinoma (HCC) globally (1). HCC is the second most common cause of cancer-related death globally, accounting for 782,000 deaths annually (2). HCC is also one of the main causes of death in cirrhosis and approximately 80-90% of cirrhotic patients have a subsequent diagnosis of HCC (3). Due to the lack of specific symptoms in the early stage, most patients are diagnosed in the middle and advanced stages, with a 5-year survival rate of approximately 18% (4).

Previous studies have reported an annual HCC incidence of 0.2–0.6% in patients without cirrhosis compared to 2.2–5% in patients with compensated cirrhosis, with the highest incidence in Asia (5, 6). Cirrhosis is a recognized risk factor for HCC, and approximately 70-80% of patients with HCC have cirrhosis. At present, antiviral therapy using nucleos(t)ide analogues (NAs), such as entecavir (ETV) or tenofovir (TDF), is the primary treatment for hepatitis B-related cirrhosis (7). Antiviral therapy inhibits HBV replication, improves liver inflammation, and reduces HCC incidence and liver-related mortality (8). The smallest HBV X protein (HBx) is required for HBV duplication and is involved in the pathogenesis of HBV-associated liver disease. Anti-HBV drugs suppress the growth of HBV-related HCC by downregulating HBx (9). Nonetheless, the risk of developing HCC persists in patients receiving NAs (10). Considering the increased incidence and high mortality of HCC, identifying high-risk patients and early surveillance are effective strategies to improve patient survival.

Currently, several HCC risk models have been developed in patients with chronic hepatitis B (CHB), including platelet age gender-B (PAGE-B) and modified PAGE-B scores (mPAGE-B) (6, 11). Although these models were externally validated and showed good performance, the majority of patients had HBV infections. These studies did not adequately predict HCC in patients with hepatitis B-related cirrhosis treated with NAs. Given that the risk of HCC can substantially increase in patients with cirrhosis, models are needed to estimate the risk of HCC in patients with cirrhosis for risk stratification. Recently, the Toronto HCC risk index (THRI), which is based on age, sex, etiology of liver disease, and platelets, was proposed to predict the risk of HCC in patients with cirrhosis of diverse etiologies (12). However, few models have estimated the risk of HCC in patients with compensated hepatitis-B related cirrhosis on antivirals in China. Overall, there is still an unmet need to apply these scores in real-world practice.

For these reasons, this study aimed to develop and externally validate a nomogram to predict HCC risk and perform risk stratification in patients with compensated hepatitis-B related cirrhosis receiving antivirals.

## Materials and methods

### Study populations

**Figure 1:**
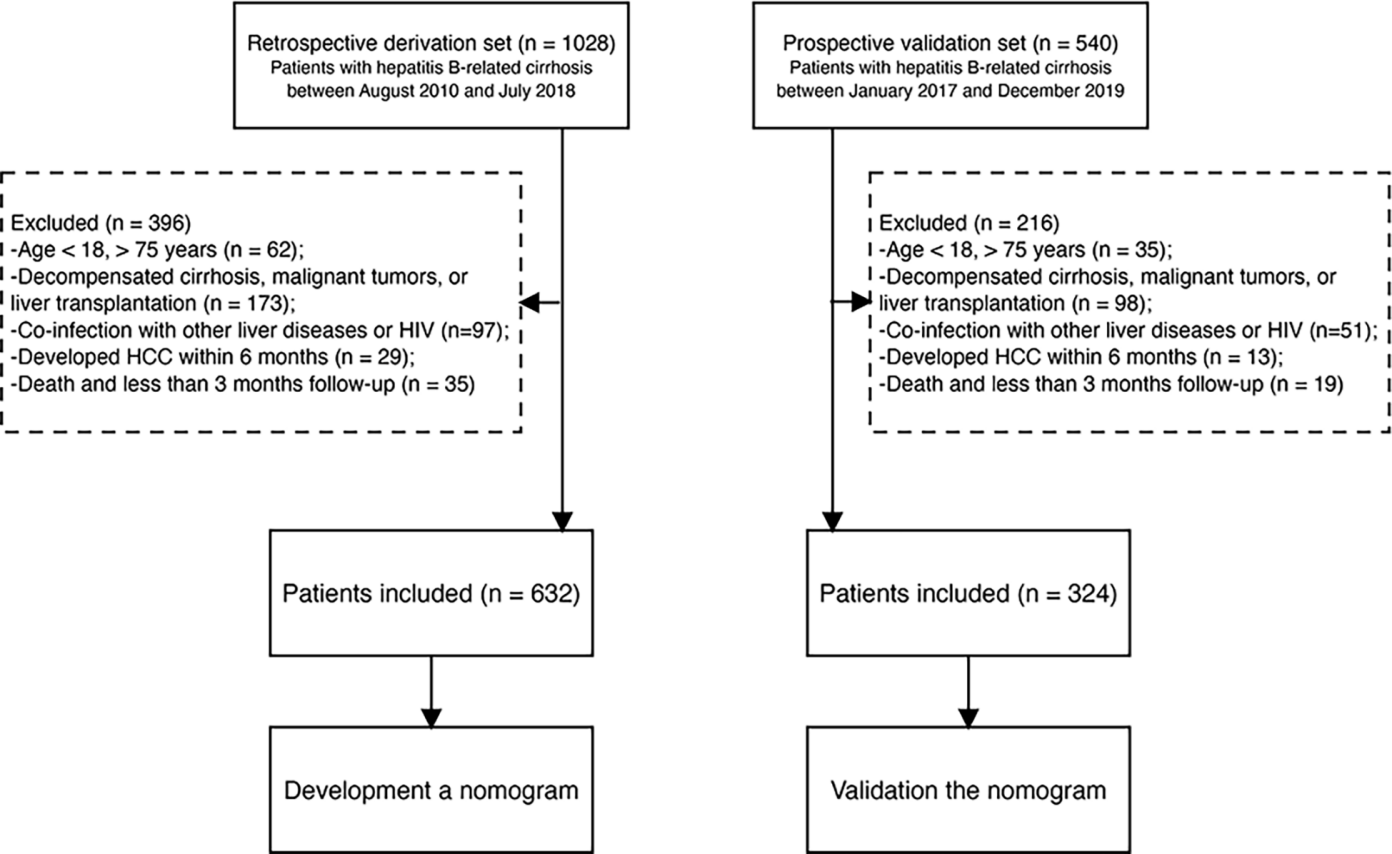
Study flow diagram. HBV, hepatitis B virus; HCC, hepatocellular carcinoma; HIV, human immunodeficiency virus.

From August 2010 to July 2018, we identified 1028 patients with compensated hepatitis-B related cirrhosis at Beijing Ditan Hospital, Capital Medical University (Beijing, China). Patients aged 18–75 years diagnosed with hepatitis-B related cirrhosis were included in the study. The inclusion criteria were as follows (1): age 18–75 years (2); patients who received ETV or TDF (3); absence of a history of HCC and organ transplant at enrollment; and (4) no previous history of decompensated cirrhosis. The exclusion criteria were as follows (1): age < 18 or > 75 years (2); co-infection with other hepatitis types (including A, C, D, and E) or human immunodeficiency viruses, or other types of liver disease (such as autoimmune hepatitis, alcoholic hepatitis, drug liver disease, fatty liver disease, idiopathic noncirrhotic portal hypertension, and genetic metabolic liver disease) (3); decompensated cirrhosis, previously diagnosed with HCC, other malignant tumors, or liver transplantation (4); development of HCC during the first 6 months; and (5) death or loss of follow-up within 3 months. After excluding patients who did not meet the inclusion criteria, 632 patients were included in the study. We also prospectively included 324 patients as an external validation cohort from Tianjin Second People’s Hospital between January 2017 and December 2019 (**Figure 1**). The date on which patients started ETV or TDF therapy was chosen as the baseline date. The outcome of the study was HCC occurrence. The study protocol was performed in accordance with the principles of the Declaration of Helsinki and was approved by the ethics committee of Beijing Ditan Hospital.

### Clinical definition and follow-up

Chronic hepatitis B was defined as HBsAg positivity for more than 6 months (13). The diagnosis of compensated cirrhosis was made following liver biopsy, endoscopy, ultrasound, or elastographic evidence of cirrhosis (14). HCC was diagnosed based on histological or radiological evidence, including computed tomography or magnetic resonance imaging (MRI), and was assessed by clinically experienced physicians (15).

Demographic characteristics and baseline data, including demographics, complications, blood routine examination, liver function, renal function, coagulation tests, HBV DNA, and alpha-fetoprotein (AFP), were recorded from a computerized database during the 24 h of enrollment. Virological response (VR) was defined as an undetectable HBV DNA load at 1 year. Continuous VR was defined as an undetectable HBV DNA load at the end of the follow-up. Neutrophil–lymphocyte ratio (NLR) was calculated as the neutrophils divided by the lymphocytes. The Model for End-stage Liver Disease (MELD), Child-Turcotte-Pugh (CTP), aspartate transaminase (AST) to platelet ratio index (APRI) score, and fibrosis-4 (FIB-4) scores were calculated according to previous studies (16–19). Every 3-6 months, routine laboratory tests and radiological examinations were performed.

### Statistical analysis

All statistical analyses were performed using SPSS version 25 (IBM, Armonk, NY, USA) and R software (version 4.1.2; R Foundation, Vienna, Austria). Continuous variables are expressed as the mean ± standard deviation or median with interquartile range (IQR) and were compared using an independent Student’s t-test or the Mann-Whitney test, as appropriate. Categorical variables are reported as numbers, percentages, and 95% confidence intervals (CIs) and were compared using the chi-square test or Fisher’s exact test.

The continuous variables were converted into classification variables to make the model simple and convenience. The cutoff points were determined based on Youden’s index for the alanine aminotransferase (ALT), AST, total bilirubin, serum albumin (ALB), NLR, platelet count, creatinine (Cr), international normalized ratio, prothrombin activity, AFP, APRI, and FIB-4 scores. The results showed that the optimal cut-off values were 40 U/L, 40 U/L, 18.8 µmol/L, 40 g/L, 1.6, 86 ×10^9^/L, 85µmol/L, 1.1, 70%, 9 ng/ml, 2, and 3.25, respectively. To identify independent risk factors influencing 3-year HCC occurrence, univariate and multivariate Cox proportional hazard regression models were used. Hazard ratios (HRs) and 95% CI were calculated. Baseline data were used to develop and externally validate the nomogram. The accuracy of the nomogram was evaluated using time dependence receiver operating curve (ROC) curves. The area under the ROC curve (AUC) was used to test the discrimination of PAGE-B, mPAGE-B, and THRI scores, which were calculated using previously published studies (6, 11, 12). We also compared the nomogram with the PAGE-B, mPAGE-B, and THRI scores at 3 years using the Delong test (20). These HCC prediction scores were calculated according to calibration curves were generated to evaluate the concordance between the risk predicted by the nomogram and the observed risk. Decision curve analysis (DCA) was used to evaluate the net clinical benefit and usefulness of this nomogram with those of the above models.

Using the 25th and 75th percentiles of the risk score in the derivation cohort, the patients were divided into low-, medium-, and high-risk subgroups. The cumulative incidence of HCC was evaluated using the Kaplan-Meier method and compared using the log-rank test. All tests were two-tailed, and a *P* value < 0.05 was considered statistically significant.

## Results

### Baseline characteristics

In the current study, 632 patients in the derivation cohort and 324 in the validation cohort were analyzed. The clinical characteristics and laboratory data of the patients are presented in **Table 1**. Among 632 patients in the derivation cohort, the median age was 47.0 (38.0–56.0) years and with male predominance (n = 456, 72.1%). The median CTP and MELD scores were 5.0 (IQR 5.0–7.0) and 8.1 (IQR 6.4–10.8), respectively. Furthermore, 608 patients (97.6%) achieved VR within the first year of therapy. Additionally, 132 (20.8%) of patients in the derivation cohort and 79 (24.4%) of patients in the validation cohort were treated with other NA(s) before ETV or TDF therapy. The median duration of follow-up was 3.3 (interquartile range: 2.4–4.9) and 2.1 (interquartile range: 1.3–3.8) years in the derivation and validation cohorts, respectively. HCC development was observed in 43 (6.8%) and 23 (7.1%) patients in the derivation and validation cohorts, respectively. No significant difference was observed in the cumulative rate of HCC incidence between the two groups.

**Table 1:** Characteristics of patients in derivation and validation cohorts at baseline.

Variables	Derivation Cohort (n = 632)	Validation Cohort (n = 324)	*P* value
Age, years	47.0 (38.0–56.0)	49.0 (39.0–57.0)	0.001
Male sex, n (%)	456 (72.1)	223 (68.8)	0.283
Family history of HCC, n (%)	50 (7.9)	49 (15.1)	0.001
Alcohol consumption, n (%)	126 (19.9)	71 (21.9)	0.179
Hypertension, n (%)	94 (14.9)	54 (16.6)	0.389
Diabetes, n (%)	79 (12.5)	50 (15.4)	0.209
HBeAg positivity, n (%)	253 (40.0)	112 (34.5)	0.115
CTP score	5.0 (5.0–7.0)	6.0 (5.0–7.0)	0.547
MELD score	8.1 (6.4–10.8)	8.5 (7.4–10.6)	0.794
ALT (U/L)	64.7 (31.5–172.2)	32.0 (22.0–66.0)	< 0.001
AST (U/L)	61.3 (30.5–188.8)	36.0 (25.6–60.8)	< 0.001
TBIL (µmol/L)	19.3 (13.0–34.0)	19.5 (13.7–30.1)	0.001
ALB (g/L)	38.6 ± 5.6	39.9 ± 6.1	0.001
WBC (×10^9^/L)	4.5 (3.3–5.7)	4.3 (3.1–5.5)	0.411
NLR	1.6 (1.2–2.3)	1.6 (1.2–2.5)	0.189
PLT (×10^9^/L)	109.0 (84.0–141.0)	116.5 (88.0–159.5)	0.136
Cr (µmol/L)	67.0 (57.0–76.0)	70.0 (58.0–77.0)	0.689
INR	1.1 (1.0–1.2)	1.1 (1.0–1.3)	0.387
AFP (ng/ml)	7.7 (3.6–26.0)	4.2 (2.5–13.5)	0.075
HBV DNA (log _10_IU/ml)	2.7 (1.1–5.8)	2.7 (1.1–6.0)	0.340
NA(s) treatment before ETV/TDF, n (%)	132 (20.8%)	79 (24.4%)	0.213
NA(s) ETV/TDF	421/211	243/81	0.783

MELD, Model for End-Stage Liver Disease; CTP, Child-Turcotte-Pugh; ALT, alanine aminotransferase; AST, aspartate aminotransferase; TBIL, total bilirubin; ALB, albumin; WBC, white blood cells; NLR, neutrophil–lymphocyte ratio; PLT, platelet count; Cr, creatinine; INR, international normalized ratio; AFP, alpha-fetoprotein; NA(s), nucleos(t)ide analogues; ETV, entecavir; TDF, tenofovir.

### Identification of risk factors

**Figure 2:**
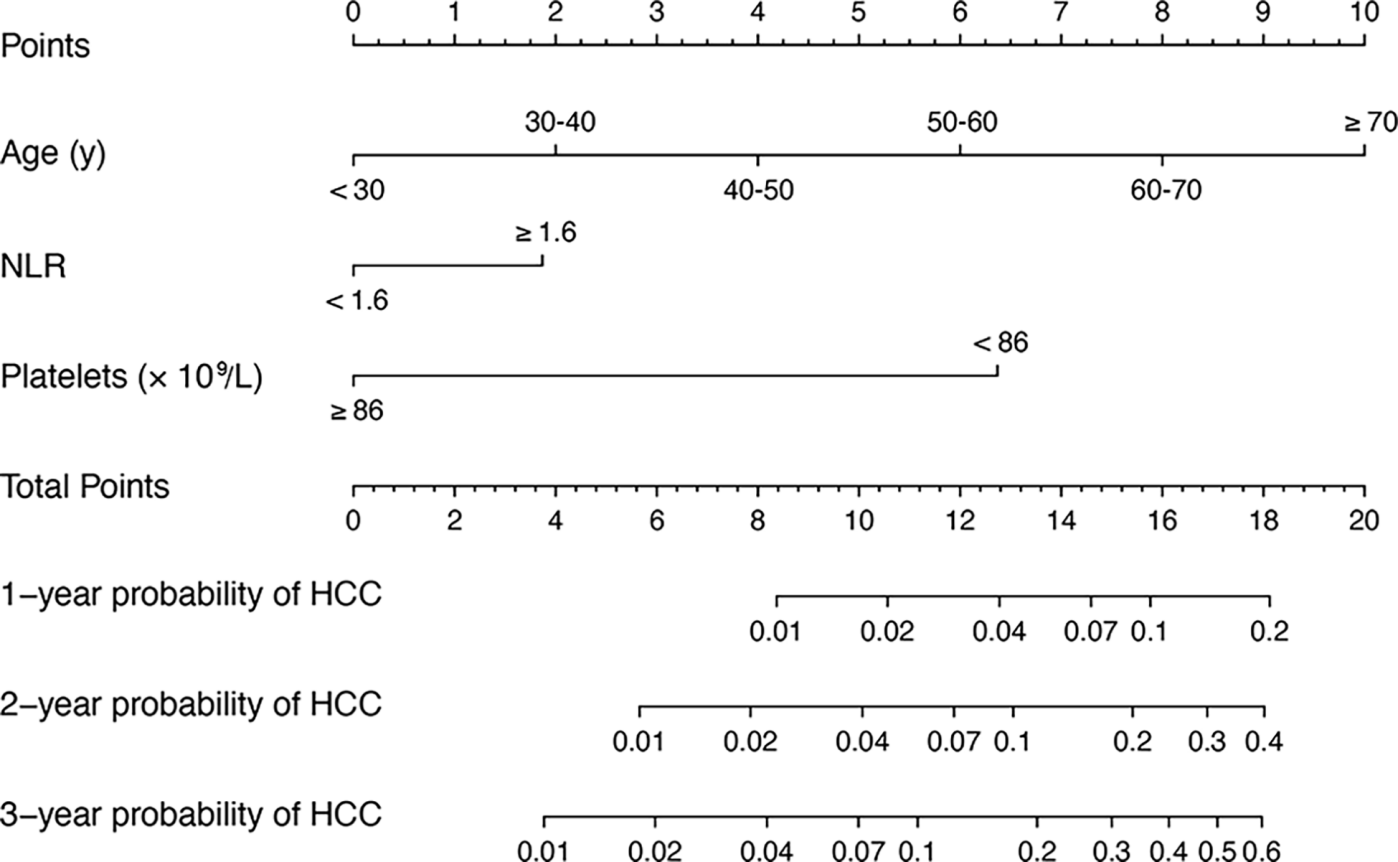
A nomogram for predicting 1, 2 and 3 years HCC risk in patients with hepatitis-B related cirrhosis. To use the nomogram, the value of an individual patient is located on each variable axis, and a line is drawn upward to determine the number of points received for the value of each variable. The sum of these numbers is located on the total point axis, and a line is drawn downward to PFS axes to determine the likelihood of HCC. HCC, hepatocellular carcinoma; hepatitis-B related cirrhosis, hepatitis B-related cirrhosis.

In univariate analysis, age, family history of HCC, ALT, AST, NLR, platelet count, and Cr were significantly associated with HCC occurrence in the derivation set (all *P* < 0.05). These significant factors were included in a subsequent multivariate Cox regression analysis, suggesting age per 10 years (HR = 1.05, 95% CI:1.02–1.07; *P* < 0.001), NLR > 1.6 (HR = 2.03, 95% CI:1.48–5.23; *P* = 0.002) and platelet count < 86×10^9^/L (HR = 4.89, 95% CI:2.51–9.51; *P* < 0.001) were independent predictors for 3-year HCC occurrence (**Table 2**). A nomogram was developed to predict the probability of HCC occurrence based on multivariate analyses, and the detailed point assignment is presented in **Table S1**. The total score was determined according to the individual scores calculated using the nomogram. Each variable is assigned a point on the top axis by drawing a line upward. The sum of these numbers is located on the total points axis, and a line is drawn downwards to the probability axis to determine the probability of HCC. The scores ranged from 0 to 20 points (**Figure 2**).

**Table 2:** Univariate and multivariate analyses of risk factors for the occurrence of hepatocellular carcinoma.

Variables	Univariate analysis		Multivariate analysis	
	HR (95% CI)	*P* value	HR (95% CI)	*P* value
Age (per 10 years)	1.07 (1.05–1.10)	0.001	1.05 (1.02–1.07)	< 0.001
Sex (male)	0.99 (0.51–1.94)	0.994		
Family history of HCC	2.43 (1.08–5.47)	0.031		
Alcohol consumption	0.64 (0.27–1.52)	0.317		
Diabetes	0.91 (0.36–2.32)	0.853		
HBeAg positivity	0.97 (0.52–1.79)	0.929		
ALT > 40 (U/L)	0.67 (0.54–0.90)	0.022		
AST > 40 (U/L)	0.50 (0.27–0.91)	0.024		
TBIL > 18.8 (mg/dl)	1.61 (0.87–3.01)	0.128		
ALB > 40 (g/L)	0.89 (0.49–1.63)	0.715		
NLR > 1.6	2.99 (1.47–6.06)	0.002	2.03 (1.48–5.23)	0.002
PLT < 86 (×10^9^/L)	6.09 (3.17–11.67)	< 0.001	4.89 (2.51–9.51)	< 0.001
Cr > 85 (µmol/L)	0.97 (0.96–0.98)	< 0.010		
PTA ≤ 70 (%)	1.31 (0.72–2.40)	0.371		
INR > 1.1	1.24 (0.68–2.26)	0.487		
AFP > 9 (ng/ml)	1.23 (0.67–2.24)	0.497		
HBV DNA > 2.7 (log_10_IU/ml)	1.74 (0.95–3.19)	0.072		
APRI score > 2	1.24 (0.75–1.95)	0.383		
FIB-4 score > 3.25	1.61 (0.84–3.10)	0.147		

ALT, alanine aminotransferase; AST, aspartate aminotransferase; TBIL, total bilirubin; ALB, albumin; NLR, neutrophil–lymphocyte ratio; PLT, platelet count; Cr, creatinine; INR, international normalized ratio; AFP, alpha-fetoprotein; APRI, aspartate transaminase to platelet ratio index; FIB-4, fibrosis-4.

### Discrimination and calibration of the nomogram

**Figure 3:**
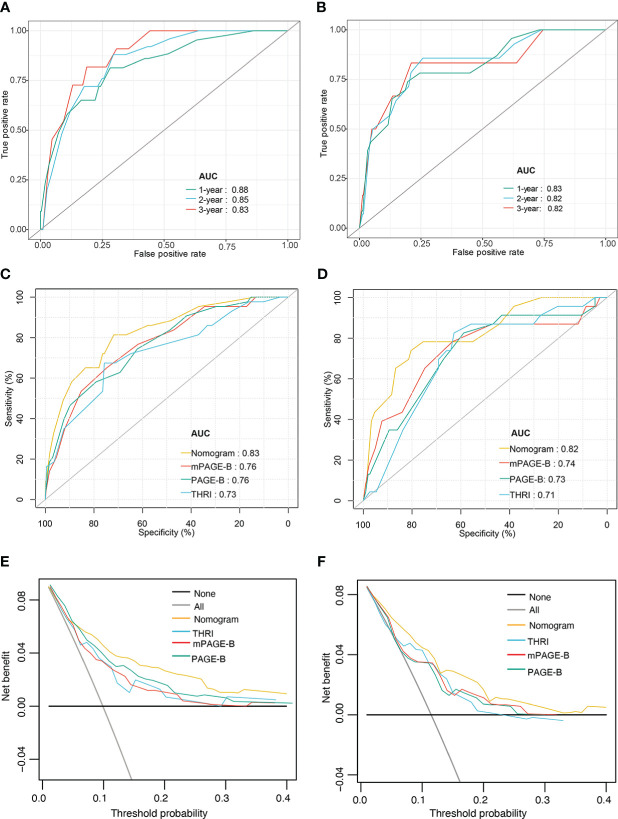
Performance of nomogram model. Time–dependent area under the ROC curve for the HCC risk prediction of patients with hepatitis-B related cirrhosis in the derivation **(A)** and the validation cohorts **(B)**; The ROC curves of nomogram, mPAGE-B, PAGE-B, and THRI in prediction of 3-year HCC occurrence in the derivation **(C)** and the validation cohorts **(D)**; Decision curve analysis for predicting HCC occurrence in the derivation **(E)** and the validation cohorts **(F)**. ROC, receiver operating characteristic; HCC, hepatocellular carcinoma; mPAGE-B, modified PAGE-B scores; PAGE-B, platelet age gender-B; THRI, Toronto HCC risk index.

As presented in **Figures 3A, B**, in the derivation cohort, the time-dependent AUCs of the nomogram for HCC occurrence were 0.88 (95% CI 0.81–0.95), 0.85 (95% CI 0.78–0.91), and 0.83 (95% CI 0.76–0.89) at 1, 2 and 3 years, respectively. In the validation cohort, the time-dependent AUCs were 0.83 (95% CI 0.63–1.00), 0.82 (95% CI 0.72–0.94), and 0.82 (95% CI 0.73–0.90) at 1, 2 and 3 years, respectively.

We compared the nomogram with estimated models for predicting HCC, including the PAGE-B, mPAGE-B, and THRI scores. The AUC of the nomogram at 3 years was significantly higher than the mPAGE-B (0.76; 95% CI 0.69–0.84), PAGE-B (0.76; 95% CI 0.69–0.84), and THRI scores (0.73; 95% CI 0.64–0.81) (all *P* < 0.05; **Figure 3C**). In the validation cohort, the nomogram provided the highest AUC (0.82; 95% CI 0.73–0.90), followed by the mPAGE-B (0.74; 95% CI 0.62–0.85), PAGE-B (0.73; 95% CI 0.62–0.84), and THRI scores (0.71; 95% CI 0.61–0.81) (**Figure 3D**).

**Figure 4:**
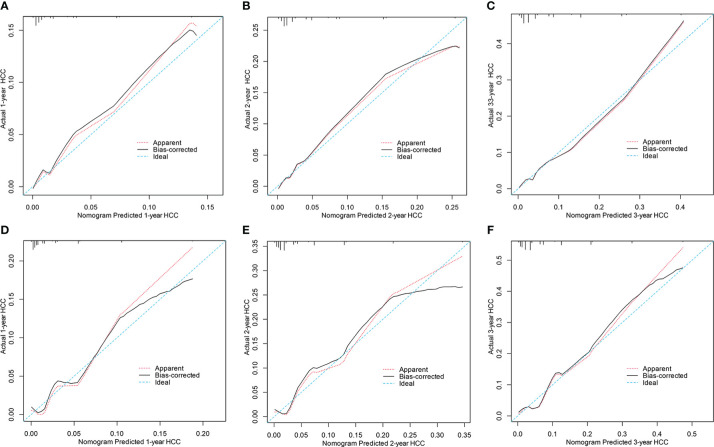
Calibration curves for predicting 1, 2 and 3years HCC probability in the derivation **(A–C)** and the validation cohorts **(D–F)**. HCC, hepatocellular carcinoma.

Compared with the PAGE-B, mPAGE-B, and THRI scores, the nomogram showed significant net clinical benefits in DCA (**Figures 3E, F**). In addition, the calibration curves illustrated that the predicted risk calibrated well with the observed risk in the derivation (**Figures 4A–C**) and the validation cohorts (**Figures 4D–F**). The nomogram had a good fit slope for predicting 1-, 2-, and 3-year HCC occurrence.

### Risk stratification for patients

**Figure 5:**
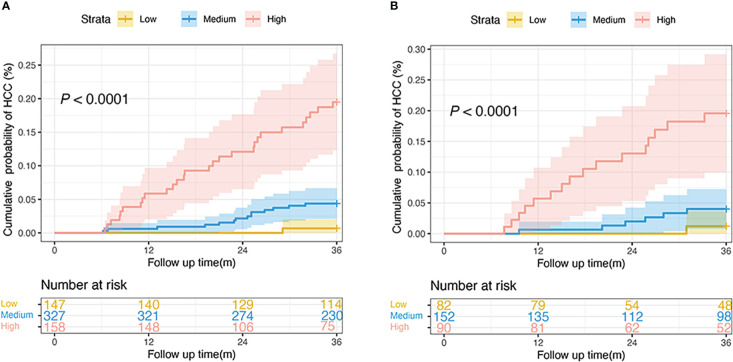
Risk stratification of HCC development according to the nomogram scores in the derivation **(A)** and the validation cohorts **(B)**. (low-risk, score: < 4; intermediate-risk, score: 4–10; high-risk: > 10; *P* < 0.0001 by log-rank test). HCC, hepatocellular carcinoma.

The 25th and 75th percentiles of the score distribution were 4 and 10 points, respectively, to stratify patients into low- (< 4), medium- (4–10), or high-risk (> 10) groups. In the derivation cohort, 147 (23.3%), low (< 4), 327 (51.7%) intermediate (4–10), and 158 (25.0%) patients had high (> 10) risk scores. Of the 324 patients in the validation cohort, 82 (25.3%), 152 (46.9%), and 90 (27.8%) had scores < 4, 4–10, and > 10, respectively. The 3-year cumulative incidences of HCC in the derivation and validation cohorts were 0.7% and 1.2% in the low-risk group, 4.3% and 3.9% in the medium-risk group, and 17.7% and 17.8% in the high-risk group, respectively (both *P* < 0.0001; **Figure 5**).

## Discussion

Recently, several studies have assessed the risk of HCC using prediction models in patients with CHB infection (21). Cirrhosis is the most important predictor of HCC; however, an accurate assessment of the risk of HCC in patients with compensated hepatitis-B related cirrhosis is limited. To address this gap, we developed and externally validated a simple nomogram to estimate the risk of HCC in patients with compensated hepatitis-B related cirrhosis and compared it with previous risk prediction scores. The nomogram, based on readily available predictors (age, platelet count, and NLR), showed effective predictive performance and stratified patients according to the estimated HCC risk into low-, medium-, and high-risk groups.

Several risk models have been developed to estimate the risk of HCC development (10, 22, 23). However, most models focus on viral factors and perform only satisfactorily in the chronic hepatitis population, thus limiting their widespread application in patients with hepatitis-B-related cirrhosis in the current era of antiviral therapy. Although PAGE-B and mPAGE-B were externally validated and exhibited good performance among patients with CHB receiving antiviral therapy, it did not specifically predict HCC risk in patients with hepatitis-B-related cirrhosis. Our nomogram is especially useful for patients with compensated hepatitis-B-related cirrhosis and achieves better performance than well-established PAGE-B and mPAGE-B scores. In the present study, the AUC values of the nomogram exceeded 0.8 in both the derivation and validation cohorts, indicating that the nomogram was accurate and reliable. A recent study presented nomograms for predicting liver-related events and the AUC predicted for HCC was lower than that of our nomogram (24). Their study was a single-center study with no validation cohort to confirm the nomograms, whereas our nomogram maintained high performance during external validation. More importantly, their study included patients with decompensated cirrhosis. However, the guidelines do not recommend monitoring for HCC in patients with decompensated cirrhosis (CTP class C) (25).

Currently, guidelines recommend biannual surveillance for all patients with cirrhosis using ultrasound, irrespective of the HCC risk (26, 27). Several studies have reported that screening for HCC in patients with cirrhosis is associated with increased survival (28, 29). An intensive screening strategy targeting patients in high-risk groups for HCC development may be more cost-effective than the current one-size-fits-all strategies, which take only cirrhosis into account (26). The presence of cirrhosis as the only criterion for monitoring is of little significance. Our findings indicate that the risk of HCC varies significantly among patients with hepatitis B-related cirrhosis, suggesting that a single screening strategy for all patients is inappropriate. It is estimated that a small number of patients with cirrhosis undergo surveillance, consistent with guidelines (30, 31). Different surveillance strategies may be proposed for different stratifications of the HCC risk. According to the risk stratification by the nomogram, patients were stratified into low (< 4), medium- (4–10), and high-risk (> 10) groups. The nomogram determined a low HCC risk in 23.3% patients in the derivation and 25.3% patients in the validation datasets. These patients could avoid the risks of incorrect diagnoses and over-treatment (physical harm from radiation and other procedures) (32). Conversely, the high-risk group had higher HCC incidence rates in the derivation and validation cohorts (17.7% and 17.8%, respectively). These high-risk patients should undergo intensive screening for early stage diagnosis, such as abbreviated MRI inspections (33). In summary, this could optimize the allocation of limited medical resources without a significant drop in effectiveness. The clinical significance of the nomogram is that it facilitates the use of readily available parameters to provide individualized counseling for patients at risk of developing HCC, which may improve compliance with surveillance proposals.

In the era of antiviral therapy, old age remains an important risk factor for the development of HCC, especially in patients with cirrhosis. In addition, the low platelet count is a well-known predictor of HCC development and prognosis (34). Platelets are related to the fibrosis stageplay a crucial role in HCC development by maintaining inflammation (35). Several studies have developed HCC risk scores; however, few have considered inflammation-related indicators. The pathogenesis of HCC is closely related to immune status and inflammatory response (36). Our and other studies have reported that a higher NLR and lower lymphocyte count are associated with a significantly higher risk of HCC in patients with hepatitis-B related cirrhosis and non-alcoholic fatty liver disease (37, 38). The immune system plays an important role in immune surveillance and controls tumor growth effects (39). Furthermore, high levels of circulating and CD8+T cells in patients with HCC have good long-term outcomes (40). Overall, we established a novel risk nomogram for HCC development among patients with hepatitis-B-related cirrhosis treated with antivirals based on NLR, which is readily obtained from routine blood examinations. The nomogram offers significant advantages over these previous risk scores of HCC, including applicability to patients with hepatitis-B-related cirrhosis in Asia, a good performance in both internal and external validation cohorts, and the ability to stratify patients into different risk groups.

To our knowledge, this is the first study to assess the performance of an HCC risk nomogram in patients with compensated hepatitis-B related cirrhosis. Risk stratification based on the nomogram is important to guide clinical practice and to optimize allocation of limited medical resources. However, this study has a few limitations. First, the nomogram was developed at a single tertiary center, and selection bias could not be avoided. However, it was validated in a prospective external cohort with a similar predictive ability. A multicenter prospective large-sample study is needed to confirm the nomogram’s prediction capacity in patients with hepatitis-B related cirrhosis. In addition, we will conduct molecular experiments in future studies to explore the relevant mechanisms. Second, HBsAg levels were not measured in some patients; therefore, the potential role of the marker could not be evaluated. Despite the lack of HBsAg levels, our nomogram showed better performance than the mPAGE-B and PAGE-B scores, which have been validated in Asian populations. Third, our study extracted data from real-world practice and we were able to clinically define cirrhosis based on ultrasound in most of the patients; therefore, some patients with early cirrhosis may be missed. However, based on our results and previous reports (41), a clinical diagnosis of cirrhosis remains important for predicting the risk of HCC in patients receiving antiviral therapy.

In summary, we developed a novel nomogram consisting of age, platelet count, and NLR to estimate HCC risk in patients with compensated hepatitis-B related cirrhosis on antivirals. Risk stratification may improve compliance with HCC surveillance, increase early detection of HCC, and reduce the harm associated with unnecessary surveillance.

## Data availability statement

The original contributions presented in the study are included in the article/**Supplementary Material**, further inquiries can be directed to the corresponding author/s.

## Ethics statement

This study was approved by the Ethical Review Committee of the Beijing Ditan Hospital (Beijing, China). Written informed consent for participation was not required for this study in accordance with the national legislation and the institutional requirements.

## Author contributions

XW and PL conceived and designed the project. KS, PL, YZ, and YB collected the data. KS and QZ analyzed and interpreted the data. KS drafted the manuscript. YZ, YB, and XZ was responsible for manuscript modification. All authors contributed to the article and approved the submitted version.
