# Supplementary material for: Development of a nomogram to predict the risk of hepatocellular carcinoma in patients with hepatitis B-related cirrhosis on antivirals
Source: Front Oncol. 2023 Feb 16;13:1128062. doi: 10.3389/fonc.2023.1128062 (PMC9978349; doi:10.3389/fonc.2023.1128062)
Supplement: Supplementary file 1 [file Table_1.docx]

Table S1. Point assignment from nomogram for predicting hepatocellular carcinoma

occurrence.

| **Risk Factor** | **Score** |
| --- | --- |
| **Age (y)** |  |
| < 30 | 0 |
| 30-40 | 2 |
| 40-50 | 4 |
| 50-60 | 6 |
| 60-70 | 8 |
| ≥ 70 | 10 |
| **NLR** |  |
| < 1.6 | 0 |
| ≥ 1.6 | 1.9 |
| **Platelets** (×10^9^/L) |  |
| < 86 | 6.5 |
| ≥ 86 | 0 |
